# The Effect of a Zeolite Addition to Modified Bitumen on the Properties of Stone Matrix Asphalt Lärmarmer Mixtures Produced as Warm Mix Asphalt

**DOI:** 10.3390/ma17235848

**Published:** 2024-11-28

**Authors:** Marta Wasilewska, Roman Pacholak, Pawel Gierasimiuk, Wladyslaw Gardziejczyk, Agnieszka Woszuk, Leslaw Bichajlo, Tomasz Siwowski

**Affiliations:** 1 Faculty of Civil Engineering and Environmental Sciences, Bialystok University of Technology, Wiejska 45E Street, 15-351 Bialystok, Poland; marta.wasilewska@pb.edu.pl (M.W.); p.gierasimiuk@pb.edu.pl (P.G.); w.gardziejczyk@pb.edu.pl (W.G.); 2 Faculty of Civil Engineering and Architecture, Lublin University of Technology, Nadbystrzycka 40 Street, 20-618 Lublin, Poland; a.woszuk@pollub.pl; 3 Faculty of Civil and Environmental Engineering and Architecture, Rzeszow University of Technology, Powstancow Warszawy 12 Avenue, 35-959 Rzeszow, Poland; leszbich@prz.edu.pl (L.B.); siwowski@prz.edu.pl (T.S.)

**Keywords:** warm mix asphalt, natural zeolite, synthetic zeolite, bitumen foaming

## Abstract

This paper presents the properties of an SMA LA (stone matrix asphalt Lärmarmer) mixture based on the polymer-modified binder PMB 45/80-55, formed by the addition of zeolites (synthetic zeolite type Na-P1 and natural zeolite—clinoptilolite). The compositions of the SMA 11, SMA 8 LA and SMA 11 LA mixtures based on modified bitumen with PMB 45/80-55 (reference mixture) or PMB 45/80-55 with Na-P1 or clinoptilolite were determined. Their resistance to permanent deformation, water sensitivity, water permeability and susceptibility to changes in texture and skid resistance during the period of use were verified. Adding zeolites reduced the production temperature by as much as 15 °C for the SMA 11 LA mixtures and 20 °C for SMA 8 LA. The addition of zeolites did not significantly affect the resistance to permanent deformation, the water permeability or the mass loss. The mixtures with clinoptilolite were resistant to the harmful effects of water, while the mixtures with Na-P1 proved more sensitive to water. Water permeability tests showed a higher permeability for SMA 11 LA compared to SMA 8 LA due to the higher nominal aggregate size. The Cantabro test showed greater particle loss for SMA 11 LA than for SMA 8 LA. A skid resistance and macrotexture analysis indicated that the SMA LA layers required special maintenance on the road due to the clogging of pores in the mix structure.

## 1. Introduction

The wearing course of roads should be durable, provide driving comfort, ensure user safety and reduce the negative impact of transport on the environment. Therefore, roadways are required to meet threshold values for parameters related to the level of transverse and longitudinal unevenness and skid resistance for a specified period of road use. This is possible through the appropriate selection of materials and the composition at the design stage of the upper surface layer. It should be noted that each country has its own policies regarding its preferred technologies for constructing pavement layers and monitoring their condition [1,2,3]. The upper layers are mainly made of asphalt mixtures. Generally, in the case of expressways, asphalt mixtures of the SMA (stone matrix asphalt), SMA LA (stone matrix asphalt Lärmarmer), BBTM (*fr.* béton bitumineuse très mince) and PA (porous asphalt) types are the most commonly used. On other roads, an asphalt mixture of the AC (asphalt concrete) type is used. Wearing courses made of asphalt mixtures are very often characterized by a texture with a negative profile of macrotextural irregularities that are characterized by a wavelength of 5.0 to 0.5 mm [4]. This is related to the high content of coarse aggregates (over 80%) in the mixture. In such cases, the microtexture has an important impact on friction, which is defined by irregularities below 0.5 mm. Therefore, in the case of these asphalt mixtures, the use of aggregate that is resistant to polishing agents is required [5,6,7,8]. The macrotexture and microtexture play important roles in skid resistance. However, the risk of slipperiness and the loss of vehicle control is always determined by wet surface conditions [5,9,10,11]. This is why awareness of the importance of the macrotexture depth and water drainage capacity at the tire/road interface is so important. It should be emphasized that a beneficial solution for reducing the risk of aquaplaning and reducing noise at the tire/surface interface is a wearing layer with a higher content of voids, at least above 10% (BBTM, PA or SMA LA) [12,13]. Despite the negative profile, water is absorbed by the channels inside the layers, which increases the contact of the dry tire with the surface in relation to layers with a closed structure. Consequently, this allows the required friction force to be obtained on wet surfaces [4].

According to the air void content and efficiency in reducing tire/pavement noise, asphalt mixtures are grouped into dense (air void content of 4–9%), semi-dense (air void content of 10–14%), semi-porous (air void content of 15–19%) and porous (air void content of >19%) categories [14]. A traditionally constructed wearing course made of a dense mixture like SMA for road surfaces that are subject to traffic is a durable solution, especially at negative temperatures. However, the problem of aquaplaning remains, which limits the generation of friction force at the contact point between the tire and the surface in slippery conditions, and the level of rolling noise generated by motor vehicles at the contact point between the tire and the surface is higher by 2–6 dB than in the case of a PA wearing course. In turn, PA layers do not guarantee satisfactory durability, especially in countries with negative temperatures in the winter [15]. Considering the need to reduce the level of rolling resistance, noise and aquaplaning, BBTM and SMA LA were designed, which is an intermediate solution between the SMA and PA mixtures. Similarly to the SMA mixture, the SMA LA mixture should be designed based on a polymer-modified binder. The content of air voids (9–12%) and the high content of coarse aggregates (approx. 80%) contribute to the “opening” of the structure and the creation of a “negative” texture for the layer made of the SMA LA mixture. This reduces noise in the contact plane of the tire with the surface and enables faster water drainage at the contact point of the tire with the SMA LA surface.

However, the SMA LA mixture is not commonly used in the construction of the wearing course in many countries. Several major studies have focused on demonstrating the noise reduction benefits of this solution. Noise level tests on SMA 8 LA-type surfaces built in Germany, Denmark and Belgium showed that these surfaces allow for a reduction in the rolling noise level of vehicles by approximately 2.0 dB compared to the reference surface made of an SMA mixture [16,17,18]. Due to the higher content of air voids, there was a concern that layers of this type would degrade faster than the traditional solution, especially in countries that have periods with negative temperatures lasting several months. A Lithuanian study conducted on the durability of SMA 8 LA and SMA 11 LA mixtures based on PMB 45/80-55 bitumen showed that the indirect tensile strength results were comparable to those obtained for traditional SMA 8 and SMA 11 mixtures. The Cantabro test results indicated that, due to the higher void content, the SMA LA layers were more susceptible to surface damage [19]. In Poland, the possibility of building SMA LA with a road bitumen binder modified with SBS (styrene–butadiene–styrene) and crumb rubber was tested. The tests proved that SMA 8 LA’s resistance to permanent deformation is similar to that of SMA 8 mixtures. But, the stiffness modules of the SMA 8 LA mixtures at low temperatures were 24% lower compared to standard SMA 8 mixtures [20]. In Belgium, the sections with a maximum aggregate size of 6.3 mm and a void content of 11% showed an extremely good resistance to raveling [17]. The state of technical knowledge in the field of research on SMA LA mixtures indicates that so far the main focus has been on research on mixtures based on polymer binder. Studies have shown that the use of this type of binder has a positive effect on the durability of SMA LA layers [18,19,20,21]. However, this requires the use of high temperatures from 150 °C to 200 °C during their production. It is not an environmentally friendly solution.

The HMA production is associated with significant emissions of harmful compounds into the atmosphere. The previous research shows that intense emissions of gases and vapors during the production of asphalt mixture at a temperature of 180 °C can be effectively reduced by lowering the temperature to 150°C [22]. Moreover, asphalt mix production at lower temperatures can reduce CO_2_ and other hazardous compounds emissions by ~40% and ~70%, respectively [23,24]. The above environmental benefits can be achieved through the use of several technologies, i.e., wax and chemical technologies [25,26,27]. The use of chemical additives reduces the viscosity of the bitumen, allowing it to be used at lower temperatures. These additives include emulsifiers, surfactants and surface tension reducers, among others. Their main advantage is that they can be easily integrated into the conventional bitumen production process and can be applied in various climatic conditions. Examples of such additives include Evotherm and Cecabase RT. Synthetic or natural waxes, such as Fischer–Tropsch or Montan waxes, are added to bitumen to lower its application temperature. The waxes work by lowering the softening point of the bitumen and modifying its viscosity. Another well-known and promising method of lowering process temperatures is the addition of zeolites to bitumen. As a result of zeolite addition to hot bitumen, the water contained in its structure begins to be released, inducing bitumen volumetric expansion leading to its foaming and viscosity reduction [28]. This process increases the workability of the asphalt mix and enhances the adhesion of the foamed bitumen [29]. Studies were also conducted on reducing the temperature of asphalt mixture production using foamed asphalt [30,31,32]. Research on WMA (warm mix asphalt) mixtures with zeolites demonstrated a 75% reduction in vapor emissions when the production temperature was reduced by 26 °C. In addition, during the incorporation process of WMA, a reduction in their emissions by approximately 90% was reported [33,34]. The production of WMA with zeolites at 135 °C allowed for a 13% reduction in energy consumption [35]. In addition, significant reductions in CO_2_ emissions of 17–37% and CO emissions of 2–6% were observed in comparison with HMA mixtures. Replacing HMA (hot mix asphalt) mixtures with WMA mixtures also means a reduction in TPM (total particulate matter) and TVOC (total volatile organic compounds) emissions by 90% and 50–70%, respectively [36].

Promising results regarding the properties of bitumen and asphalt mixes were obtained using natural zeolite clinoptilolite [34,37,38,39,40], whereas Woszuk et al. [40,41] demonstrated that synthetic zeolites produced from waste materials can also be successfully used in WMA technologies. The idea behind the use of WMA technology is that the production of an asphalt mixture at a reduced temperature will allow obtaining a product with comparable or improved physical and chemical properties compared to HMA technology [42]. As the previous studies indicate, compared to HMA mixtures, the use of asphalt foaming technology with zeolites allows for improving the compactability [43,44] and resistance to permanent deformation of the obtained asphalt mixtures. However, in order to obtain satisfactory resistance to water and frost, it is recommended to apply hydrated lime as an adhesive agent [43].

Being aware of the numerous benefits resulting from the construction of SMA LA wearing surfaces, a decision was made to conduct research to verify the possibility of using a binder modified with zeolite additives. These additives are intended to lower the temperature during production. This would allow us to produce a durable, safe and environmentally friendly surface. Therefore, selected properties of SMA LA mixtures with the addition of two types of zeolites were assessed and compared with reference mixtures.

## 2. Research Program

**Figure 1:**
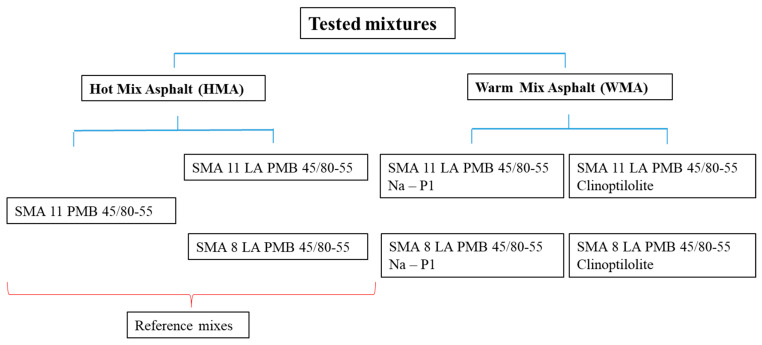
Tested mixtures.

The object of this study was to examine SMA LA mixtures with the addition of natural zeolite—clinoptilolite and synthetic zeolite—Na-P1, used to lower technological temperatures in the production process. The mixtures were differentiated by the maximum grain size of the coarse aggregate. The compositions of the SMA 8 LA and SMA 11 LA mixtures were designed. A modified PMB 45/80-55 binder was used for the mixtures, to which a specified amount of zeolite was added. To determine the effect of the zeolite addition on the properties of individual SMA LA mixtures, reference mixtures were included in the program. These were SMA 11 LA and SMA 8 LA mixtures based on PMB 45/80-55 modified bitumen without any zeolite addition, classified as HMA. To determine the benefits of using SMA LA mixtures both with and without zeolites, a decision was made to include a third reference mixture—the SMA 11 mixture traditionally used for road surfaces. The tested mixtures are shown in Figure 1.

The scope of this study included determining the following:

- Temperatures in the compaction process of the SMA LA mixtures with the addition of zeolite at optimal ranges of volume parameters;
- Water sensitivity according to [45];
- Rutting resistance according to [46];
- Particle loss using Cantabro test according to [47];
- Water permeability according to [48] and Polish National Specification [49];
- Macrotexture according to [50];
- Skid resistance according to [51].

## 3. Materials and Methods

### 3.1. Materials

#### 3.1.1. Modified Bitumen

The conventional PMB 45/80-55 binder modified with SBS was used in the study. PMB is produced according to [52] and the Polish National Annex to this standard. In order to lower the technological temperature, it was modified by adding natural zeolite—clinoptilolite and artificial zeolite—Na-P1. Clinoptilolite (ZN-C) in the form of zeolitic tuff was derived from the Sokyrnytsya deposit (Transcarpathian region, Ukraine). Synthetic zeolite Na-P1 was produced at the Lublin University of Technology from fly ash by hydrothermal reaction. The structure of the clinoptilolite consists of two-dimensional channels formed by eight-membered rings of 4.1 × 4.1 Å and 10-membered rings of 2.8 × 4.8 Å. The structure of Na-P1 is formed by two four-membered rings forming an eight-membered channel with dimensions of 3.1 × 4.5 Å and 2.8 × 4.8 Å.

Before the modification process, the zeolites were pre-saturated with water. The water content in dry zeolite Na-P1 was 75% and, in clinoptilolite, 25%. Water introduced into the crystal structure of zeolites in this way can be released in the temperature range from 100 to 450 °C. As a result, microfoam is formed in the modified bitumen. Zeolites at a rate of 5% (by weight of the binder) were added to PMB 45/80-55 and preheated to 175 °C. The temperature of modification and the amount of introduced water show a significant effect on the quality and quantity of the generated foam. The components were mixed by hand for 1 min until foam was formed, and then the samples were placed back in the oven at 175 °C for 30 min for stabilization. Table 1 shows the properties of the modified bitumen used.

#### 3.1.2. Asphalt Mixtures

Asphalt mixtures were designed according to [59] and Polish National Specification [49]. The compositions of SMA 11, SMA 8 LA and SMA 11 LA mixtures based on PMB 45/80-55 produced as HMA and on the basis of PMB 45/80-55 with Na-P1 and with clinoptilolite were determined. Fine and coarse aggregates obtained from gabbro rock were used. The physical properties of coarse aggregate are given in Table 2.

Mixtures depending on the maximum aggregate size were designed with the same or similar binder content and aggregate particle size distribution (Table 3).

To determine the volumetric parameters, the SMA 11 mix was compacted in a Marshall compactor with 50 blows on each side in accordance with [65]. Due to the higher content of voids in the SMA LA mixes compared to SMA 11, they were compacted in a gyratory compactor for 20 revolutions in accordance with [66]. Impact from a compactor would damage the structure of this type of mix.

In the case of the reference mixes, the heating temperature of the mineral mix was 195 °C and PMB 45/80-55—175 °C, and the compaction temperature of the mixes was 145 °C. Based on the conducted tests of the bulk density *ρ_bsea_* (sealed samples) and the density of the asphalt mix *ρ_mv_*, the content of voids in the SMA 8 LA and SMA 11 LA mixes were determined. In the case of mixtures with particular zeolites, the temperature of mineral component heating was reduced to the bitumen temperature of 175 °C. The optimum compaction temperature of asphalt mixtures was determined experimentally by conducting tests on samples compacted in the gyratory compactor at temperatures of 130 °C, 125 °C and 115 °C. It was found that the volumetric parameters of the mixtures SMA 8 LA with clinoptilolite and Na-P1 are comparable with the values obtained for the reference mixture SMA 8 LA at 125 °C, while the parameters of the mixtures SMA 11 LA with clinoptilolite and Na-P1 are comparable with the reference mixture SMA 11 LA at 130 °C. Volumetric parameters of each mixture are presented in Table 4, Table 5 and Table 6.

### 3.2. Methods

#### 3.2.1. Water Sensitivity Test

A water sensitivity test of asphalt mixtures was carried out according to [45] (method A) and Polish National Specification [49]. Ten specimens for each mixture were divided into two sets: “dry set” and “wet set”. Specimens from the “dry set” were conditioned at a temperature of 20 ± 5 °C. Specimens from the “wet set” were saturated with distilled water under vacuum (6.7 ± 0.3 kPa, 30 min) and left in water for 30 min under atmospheric pressure. Then, specimens were conditioned in water at a temperature of 40 °C for 72 h. In the next step, plastic-foiled specimens were frozen at −18 °C for 16 h and then placed in water at a temperature of 25 °C for 24 h. Finally, the ITS (indirect tensile strength) test in accordance with [70] on both sets of specimens was conducted. The *ITSR* values were calculated according to Equation (1):

(1) $$ITSR=\frac{{ITS}_{w}}{{ITS}_{d}}\cdot 100,\%$$

where

*ITS_w_*—mean values of ITS for specimens from ”wet set” [kPa];
*ITS_d_*—mean values of ITS for specimens from “dry set” [kPa].

#### 3.2.2. Rutting Test

Rutting resistance of SMA mixtures was conducted according to [46] in a small-sized device (method B), in air and in a large-sized device. Two specimens from each mixture were prepared. Before testing, specimens were conditioned at 60 °C for 4 h. The apparatus consists of a loaded wheel that repeatedly passes over the test specimen. The wheel load was 700 N, and the frequency was 26.5 ± 1.0 load cycles per minute. The test was performed at 60 °C. Evaluation of resistance to rutting is made based on the following:

− RD_AIR_ (rut depth) after 10,000 cycles [mm];
− PRD_AIR_ (proportional rut depth) after 10,000 cycles as a percentage of the specimen thickness [%];
− WTS_AIR_ (wheel-tracking slope) [mm/10^3^ load cycle].

The tests in the large-sized device were also carried out on two independent specimens. The conditioning of the samples and the test were carried out at a temperature of about 60 °C. The pressure in the pneumatic tire was about 600 kPa, the load of the rolling wheel was 5000 N and the wheel displacement frequency was 1.0 Hz. Evaluation of resistance to rutting is made based on the mean PiLD (proportional rut depth) values calculated from two specimens.

#### 3.2.3. Horizontal and Vertical Water Permeability

Horizontal and vertical water permeability test was carried out according to the standard [48]. SMA 11 specimens were prepared by Marshall compactor according to the standard [65] (2 × 50 blows) and SMA LA specimens were prepared in the gyratory compactor to a height of 63.5 ± 2.5 mm in accordance with the standard [66].

To determine the water permeability in the vertical direction, the sample was placed in the device and a water flow at 300 ± 1 mm was allowed. To prevent leakage of water along the tube wall, the tube was covered with a rubber membrane and a pressure of 50 kPa was applied. The test temperature should oscillate between 15 and 25 °C. It takes at least 10 min for the water to flow through the sample to saturate the specimen and to remove enclosed air. An empty vessel of mass m_1_ was then placed and the water was allowed to flow for 1 min. After 1 min, the amount of water entering the vessel m_2_ was determined and the vertical water permeability was calculated from Equation (2):

(2) $$Q_{v}=\frac{m_{2}-m_{1}}{60}\cdot 10^{-6},[m^{3}/s]$$

Darcy’s formula for calculating the vertical water permeability of samples takes the form of Equation (3):

(3) $$K_{v}=\frac{4\cdot Q_{v}\cdot l}{h\cdot \pi \cdot D^{2}},\;[m/s]$$

where

*l*—sample height [m];
*h*—water column height [m];
*D*—diameter of the sample [m].

To determine horizontal water permeability, the sample was sealed at the bottom with paraffin to reduce the possibility of vertical penetration. A plastic tube was glued with silicone to the top surface of the sample. The sample prepared in this way was placed in a vessel and left for 10 min. After this time, the plastic tube was filled with water so that its column had a constant height of 300 ± 1 mm. After a minute of free seepage, the mass of water m^2^ that would flow through the side walls of the sample into the vessel of mass m_1_ in t = 60 s was measured.

Horizontal water flow was determined according to Equation (4):

(4) $$Q_{h}=\frac{m_{2}-m_{1}}{60}\cdot 10^{-6},\;[m^{3}/s]$$

The horizontal water permeability of the sample was determined according to the modified Darcy Equation (5):

(5) $$K_{h}=\frac{Q_{h}\cdot l}{0.3\cdot (\pi \cdot D\cdot l)},\;[m/s]$$

where

*l*—height of the sample [m];
*D*—diameter of the sample [m].

#### 3.2.4. Cantabro Test

In this test, the breakdown of compacted specimens is measured using the Los Angeles machine according to [47]. The percent of weight loss (particle loss—*PL*) is an indication of durability and relates to the quantity and quality of the bitumen binder. Specimens for testing should be compacted to a height of 63.5 ± 2.5 mm. After conditioning at 25 °C, each specimen with an initial mass of *W*_1_ was placed in a Los Angeles machine drum without metal balls. After 300 revolutions of the drum at a speed of 30–33 rpm, the mass of specimen *W*_2_ was determined again. The degradation of the asphalt mixtures was calculated based on Equation (6):

(6) $$PL=\frac{W_{1}-W_{2}}{W_{1}}\cdot 100,[\%]$$

where

*PL*—value of particle loss [%];
*W*_1_—initial specimen mass [g];
*W*_2_—final specimen mass [g].

#### 3.2.5. Macrotexture and Skid Resistance Test

**Figure 2:**
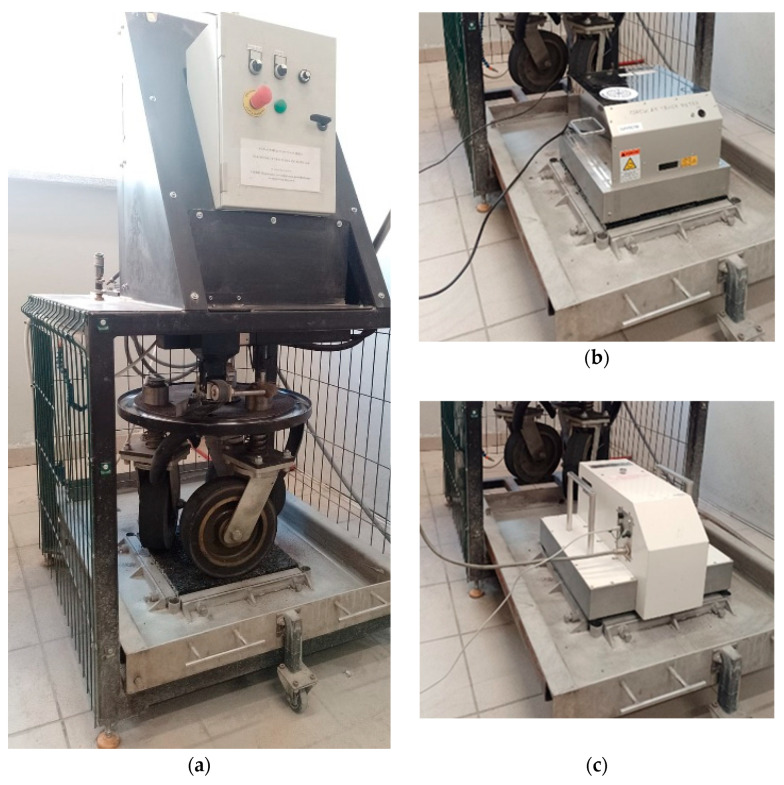
(**a**) A device simulating the polishing process, (**b**) measurement of macrotexture using the CTM device, (**c**) measurement of the friction coefficient using the DFT device.

To determine the changes in macrotexture and the skid resistance, the specially prepared samples in the form of plates were subjected to surface wear simulation under laboratory conditions. A device developed at Bialystok University of Technology was used. The test specimens had dimensions of 400 × 400 × 40 mm. The test consisted of simulating phenomena occurring in actual traffic conditions. For this purpose, a machine consisting of three rubber wheels moving on the surface of the specimen using a rotating disk was used (Figure 2). The wheels were moving at a speed of 30 ± 2 rpm. The entire test consisted of two 3-h phases. In the first phase, water and coarse emery (300/600 µm) were fed continuously to the surface of the sample on which the wheels were moving, and, in the second phase, water and emery flour (<53 µm) were fed. The macrotexture changes were determined based on measurements using a CTM (Circular Texture Meter) laser profilometer in accordance with [50]. The skid resistance changes were assessed based on measurements with a DFT (Dynamic Friction Tester) device in accordance with [71].

The CTM determines the unevenness profile based on which parameters characterizing the macrotexture MPD (Mean Profile Depth) and RMS (Root Mean Square) are calculated. The profile is measured by a CCD (Charge-coupled Device) laser displacement sensor that moves around the circumference of a circle with a radius of 142 mm. The resolution of the device is 0.87 mm. The MPD parameter measured before and after each phase in a trace of polishing wheels was adopted for the analyses.

The DFT consists of two disks that are connected to each other by a spring and a sensor. There are three rubber sliders mounted on the lower disk. Disks rotating at a speed of 80 kph are lowered to the pavement and the friction coefficient is measured. The DFT(E) 5.0 software determines the friction coefficient DFT20 at a slip speed of 20 kph. The DFT20 measurement was performed before the process and at the following polishing times: 30 min, 60 min, 120 min, 180 min, 210 min, 240 min, 300 min and 360 min.

## 4. Results and Discussion

### 4.1. Water Sensitivity

**Figure 3:**
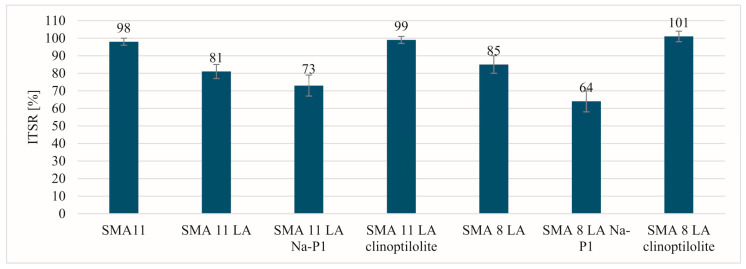
The ITSR results.

Figure 3 presents ITSR results. The highest values (above 95%) were obtained for the reference mixture SMA 11 and the mixtures SMA LA with clinoptilolite. The mixtures with Na-P1 have the lowest ITSR results: SMA 11 LA—73% and SMA 8 LA—64%. The reference mixtures with SMA 8 LA and SMA 11 LA have comparable ITSR results ranging from 80 to 85%. The ITSR results above 80% are satisfactory and meet the criteria in many European countries [38]. It can therefore be assumed that these asphalt mixtures are insensitive to water.

**Figure 4:**
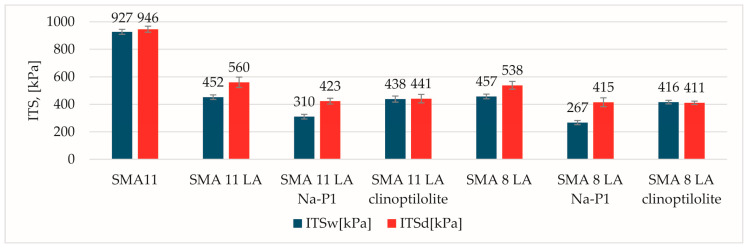
The ITS results.

The significant differences between the SMA 11 reference mixture traditionally used for wearing courses and the SMA LA mixtures are visible in the ITS values (Figure 4). The SMA 11 had the highest values irrespective of specimen conditioning. They are almost twice as high as the values obtained for the other mixtures. It was unexpected that wet and dry specimens of SMA LA with clinoptilolite would be comparable. This may be due to water retention in the pores of the saturated sample and the pressure it generates during testing. The lowest ITS values were obtained for the SMA LA mixture with Na-P1. In addition to this, it was not noticed that increased nominal aggregate size resulted in a decrease in the ITS values.

Existing data from the literature indicate that asphalt concrete with added zeolites designed to the base layer was also characterized by slightly lower water resistance [72], especially with the application of water-soaked zeolites [38]. In contrast, an increase in the ITSR index was observed in asphalt concretes prepared for the surface layer [73]. Water resistance in zeolite-soaked WMA was also improved by adding 1.5% hydrated lime, while standard release agents were found to be ineffective [74].

In general, the SMA LA mixtures performed worse than the SMA mixture in terms of indirect tensile strength. It was proved in other research that these results are related to air void content [13]. However, the increased void content in comparison to traditional solutions is crucial to reducing noise and the risk of aquaplaning. Therefore, further tests were performed to determine the durability of wearing layers made of SMA LA mixture with the zeolite additions.

### 4.2. Resistance to Rutting

**Figure 5:**
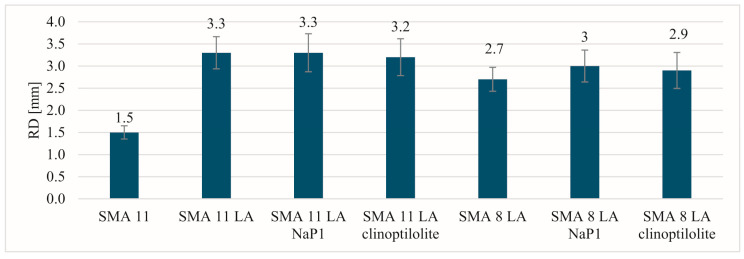
The rut depth results.

**Figure 6:**
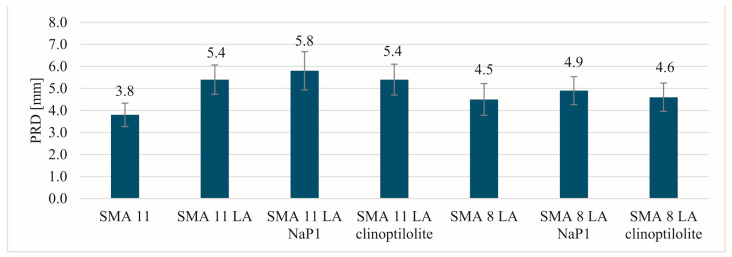
The proportional rut depth results after 10,000 cycles.

**Figure 7:**
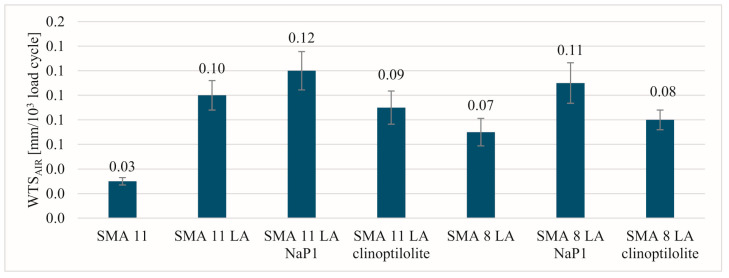
The wheel-tracking slope results.

**Figure 8:**
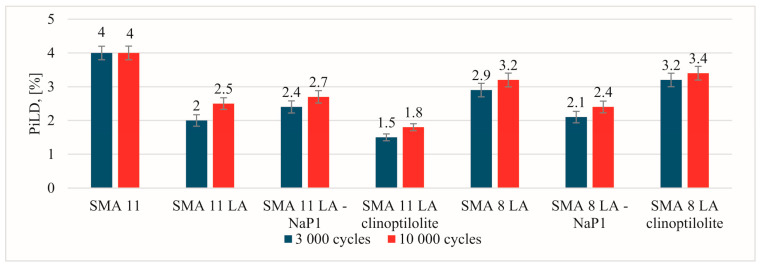
The proportional rut depth results obtained from a large-scale device.

The most beneficial results of the parameters indicating resistance to permanent deformation in a small-sized device were recorded for the SMA 11 mixture (Figure 5, Figure 6 and Figure 7). Comparing the values of individual parameters obtained for SMA 11, it can be seen that they are significantly lower than in the case of SMA LA. In addition, the SMA LA mixtures show differences in the results related to the nominal aggregate size. The results for the SMA 8 LA mixtures were slightly lower than those obtained for the SMA 11 LA mixtures. Based on these results, it may be seen that the SMA LA layers will be deformed in the tracks of car wheels. Similar conclusions were obtained in studies conducted using the same method but on samples BBTM 5 and 8. It was found that the worse results are related to the content of voids in such mixtures [13]. However, experience in other European countries indicates that wearing courses made from semi-dense, semi-porous and porous mixtures are resistant to permanent deformations [75]. To check them at the stage of designing the composition in the laboratory, it is recommended to use a large-sized device. That is why these tests were conducted. Based on the test results obtained from the large-scale device, the SMA LA mixtures are more resistant to rutting than the traditionally used SMA 11 (Figure 8). The lowest values of the PiLD were obtained for SMA 11 LA with clinoptilolite. It was 1.5% after 3000 cycles and 1.8% after 10,000 cycles. Slightly higher values were obtained for SMA 11 LA, SMA 11 LA with Na-P1 and SMA 8 LA with clinoptilolite. No effect of the addition of the particular zeolites on the resistance to permanent deformation was noted. It should be noted that the results of both reference mixtures and those with the addition of zeolites are satisfactory.

### 4.3. Water Permeability

Table 7 and Table 8 present the results of vertical and horizontal water permeability. Significant differences due to the nominal size of the aggregate were observed. SMA 11 LA mixtures are characterized by greater permeability in both directions The results relate to the spaces created by interconnected voids that allow water to flow. Although the mixtures have a comparable void content, wider capillaries are formed in SMA 11 LA than in SMA 8 LA.

The addition of natural zeolite slightly reduces the vertical water permeability (K*_v_* = 4.95·10^−4^ m/s) compared to the reference SMA 8 LA mixture (K*_v_* = 5.05·10^−4^ m/s). This indicates that the addition of clinoptilolite results in a more compact mixture structure. Vertical water permeability of specimens with Na-P1 achieved a value equal to K*_v_* = 4.98·10^−^⁴ m/s, which is almost identical to that of the samples with clinoptilolite. Both additives have a similar effect, as they slightly reduce vertical water permeability, but the differences are not very significant. Both additives produce similar levels of vertical water permeability in each of the SMA 11 LA mixtures. Therefore, the type of additive has no significant effect on the vertical water permeability, and this effect decreases with increasing nominal aggregate size.

Clinoptilolite reduces the horizontal water permeability to K_h_ = 1.02·10^−3^ m/s compared to the reference SMA 8 LA mixture (K_h_ = 1.09·10^−3^ m/s). This shows that clinoptilolite can also create a denser structure or change the internal pore structure in a way that reduces horizontal water permeability. Similarly, but slightly less, Na-P1 reduces horizontal water permeability to K_h_ = 1.04·10^−3^ m/s. In SMA 11 LA mixtures, both additives again show no significant effect, as the K_h_ values remain like those of the reference mixture. Zeolites do not change the horizontal water permeability for mixtures with a higher nominal aggregate size.

The SMA 8 LA mixture shows sensitivity to the addition of zeolites, which slightly reduces the horizontal and vertical water permeability, while the water permeability of the SMA 11 LA mixture remains practically unchanged.

### 4.4. Raveling Resistance

**Figure 9:**
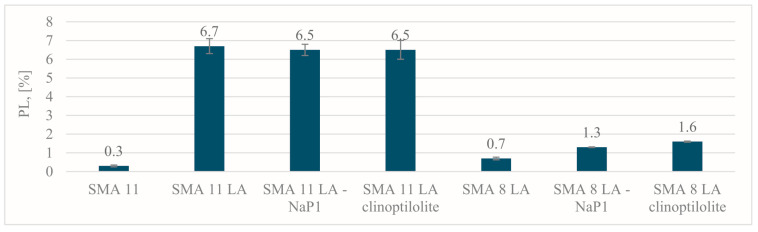
The Cantabro test results.

Figure 9 shows the PL results describing the resistance to mass loss. The more interconnected air voids in a mixture, the less resistant it is to mass loss. This is closely related to the gradation of the mineral mixture—the larger the nominal maximum aggregate size in the mineral mixture, the lower the resistance to particle loss. Samples of the SMA 11 LA mixture show a significantly higher grain loss compared to the SMA 8 LA mixture.

In SMA 11 LA mixtures the particle loss values are very similar and the difference between SMA 11 LA without additives (PL = 6.7%) and SMA 11 LA with Na-P1 and clinoptilolite (PL = 6.5%) is small. It can be concluded that zeolites in the SMA 11 LA mixture do not have a significant effect on the resistance to mass loss improvement. However, the addition of zeolites did not worsen this parameter.

In the SMA 8 LA mixtures, although the observed differences are noticeable, they are not significant. The reference mixtures obtained a particle loss (PL = 0.7%) and, after the addition of zeolite Na-P1, this parameter increased to PL = 1.3%. An even greater increase in particle loss was observed with the addition of clinoptilolite (PL = 1.6%). Although the addition of zeolites slightly reduces resistance to particle loss, these are not very significant changes and the mixtures still demonstrate high resistance to raveling.

### 4.5. Macrotexture and Skid Resistance

**Figure 10:**
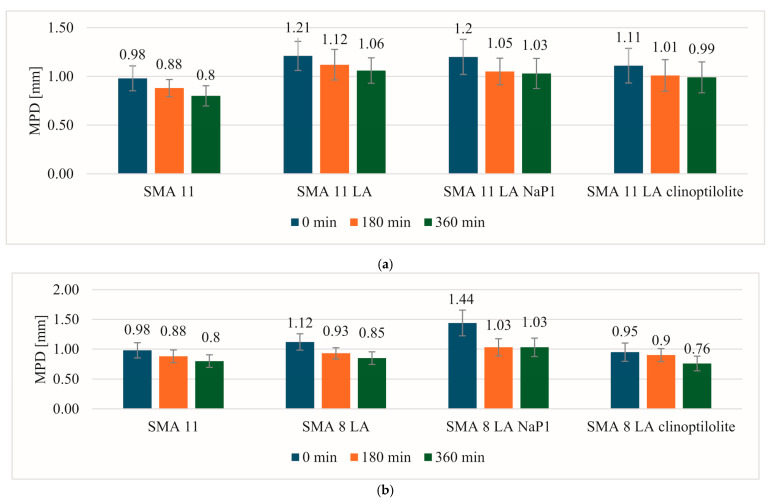
Comparison of the change in the MPD value of the reference mixtures and (**a**) SMA 11 LA mixtures, (**b**) SMA 8 LA mixtures.

Figure 10 presents the change in the MPD value depending on the wear process. The MPD parameters of each specimen were the highest before the test. It should be noted that higher values were recorded for specimens made of SMA 11 LA and SMA 8 LA mixtures. The exception is SMA 8 LA with Na-P1 before the test. Although the mixtures with a maximum grain size of 8 mm had the same mineral mixture composition, the MPD value was very high (1.44 mm). This may be related to the plate compaction process. However, in this case, the decrease in MPD value was the highest and amounted to 0.41 mm after the first phase (3 h). In the case of the remaining mixtures, the values decreased from 0.19 mm to 0.05 mm depending on the mixture. The MPD of SMA 8 LA specimens with clinoptilolite decreased by 0.14 mm after the second phase. However, in other mixtures, it was not higher than 0.80 mm in relation to the value obtained after the first phase. The surface of the reference SMA 11 mixture showed slight decreases after each phase by about 0.10 mm. Before the start of the test, it showed the lowest result. After the test, the MPD value of this mixture is comparable to that of SMA 8 LA, but lower than that of SMA 11 LA.

Based on the observation of the surface of the SMA LA specimens, it was noticed that the decrease in MPD is related to the clogging of the spaces between aggregate grains by the materials used to simulate the abrasion and polishing phenomenon. Even though water was dosed during the test and the plates were carefully washed after the completion of the phases, the sludge penetrated deeply into the structure of the mixtures. This is also confirmed by the texture profiles. Similar hazards occur in actual traffic conditions.

**Figure 11:**
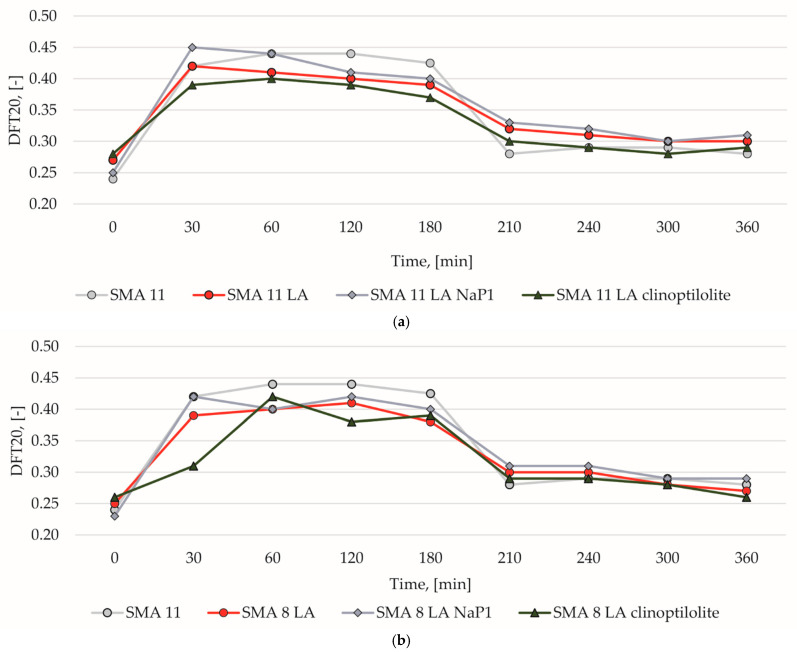
Comparison of the change in the DFT20 value of the reference mixtures and (**a**) SMA 11 LA mixtures, (**b**) SMA 8 LA mixtures.

Coarse and fine corundum emery should be used to simulate phenomena occurring under the influence of car traffic and the presence of contamination on the road surface. They have a major impact on changes in microtexture. The DFT20 is used as a surrogate for microtexture. Figure 11 shows the changes in this parameter during the test. The values are low before the test starts and they are due to the presence of binder on the aggregate surface. Low skid resistance on new asphalt mix surfaces is a known phenomenon. For this purpose, in the process of compacting the wearing course, a 2/4 mm fraction aggregate is applied to its surface, which roughens the surface. Its purpose is to protect the road surface from slipperiness in the initial period of its use. Grains from the roughening break off and accelerate the removal of the binder film from the coarse aggregate grains of the mixture under the influence of car traffic. As a result the friction coefficient increases. However, coarse aggregate cannot be used on SMA LA surfaces as it may destroy their structure. Therefore, there is a possibility of loss of noise reduction and aquaplaning prevention capabilities.

However, in actual conditions, coarser contaminations and those corresponding to the dust fraction are also present. The first of them corresponds to the abrasion phenomenon, which manifests itself by the matting of the aggregate surface and the formation of grooves. On the other hand, dust contributes to the polishing of the surface [21]. Abrasion and polishing occur with varying intensity depending on the atmospheric conditions in actual traffic conditions. To simulate them in the laboratory, two phases were distinguished. In the abrasion phase, the SMA11 mixture is characterized by the highest DFT20 values. SMA 11 LA and SMA 8 LA have a similar course of friction coefficient changes. In both cases, SMA LA mixtures achieved higher results when clinoptilolite was added to the bitumen. A similar tendency occurs in the polishing phase. However, mixtures with grains up to 11 mm are characterized by slightly higher values compared to SMA 8 LA. This may be due to the larger aggregate surface area on which the friction test was performed. The final results of the SMA 11 LA and SMA 8 LA are comparable and no significant influence of the addition of zeolites on the final test result can be observed.

## 5. Conclusions

The purpose of this study was to verify the feasibility of using zeolites in the form of clinoptilolite, synthetic zeolite Na-P1 in the polymer-modified binder PMB 45/80-55 and to evaluate the effect of these additives on the properties of SMA LA (Stone Matrix Asphalt Lärmarmer) asphalt mixtures produced in warm mix asphalt (WMA) technology. Results allow us to formulate the following conclusions:

1. The addition of zeolites allows for a significant reduction in the technological temperature of SMA 11 LA mixtures by up to 15 °C compared to the traditional HMA (hot mix asphalt) technology. In the case of SMA 8 LA, the temperature reduction was 20 °C, resulting in energy savings and reduced greenhouse gas emissions. Additionally, the aggregate heating temperature was lowered to the bitumen temperature.
2. SMA LA mixtures modified with zeolites demonstrate adequate water resistance, with clinoptilolite showing higher ITSR values (above 95%), indicating lower sensitivity to water. In contrast, SMA LA with Na-P1 had lower ITSR values (73% for SMA 11 LA and 64% for SMA 8 LA), suggesting slightly higher water sensitivity. However, most of the mixtures met the water resistance criteria applicable in many European countries, with ITSR values exceeding 80%.
3. To determine the rutting resistance of SMA LA mixtures in laboratory conditions, it is recommended to use a large-sized device. SMA LA mixtures showed slightly better rutting resistance compared to the conventional SMA 11. The most favorable PiLD values obtained for SMA 11 LA with clinoptilolite were 1.3% after 3000 cycles and 1.8% after 10,000 cycles. For other variants, such as SMA 11 LA and SMA 11 LA with Na-P1, the values were 2% and 2.5% after 3000 and 10,000 cycles, respectively. SMA 8 LA with clinoptilolite recorded 2.9% after 3000 cycles and 3.2% after 10,000 cycles. The addition of zeolites did not significantly affect the resistance to permanent deformation of the reference and zeolite-modified mixtures.
4. Water permeability tests showed that SMA 11 LA mixtures had higher permeability than SMA 8 LA due to the larger aggregate size. The addition of zeolites had little effect on vertical water permeability, while clinoptilolite slightly reduced horizontal permeability, suggesting a denser mix structure.
5. The Cantabro test showed that SMA 11 LA mixtures had higher particle loss values compared to SMA 8 LA due to larger aggregate size. The reference mixture for SMA 11 LA showed a weight loss of 6.7%, while the mixtures with Na-P1 and clinoptilolite showed a similar loss level of 6.5%. All mixtures that exhibited good resistance to the raveling and zeolite addition did not significantly affect the PL parameter.
6. All SMA LA samples showed a decrease in MPD due to higher void content compared to traditional SMA 11 mixtures, indicating potential maintenance needed in real road conditions: modification of maintenance procedures by introducing flushing with higher water pressure, which may help to remove contaminants. New wearing courses made of SMA LA mixture without the removed asphalt film on coarse aggregate demonstrate slippery surfaces. DFT20 friction coefficients are at a comparable level in the range of 0.26 to 0.29 after the completion of the abrasion and polishing processes in slab polishing.

In conclusion, the use of zeolites in bitumen in SMA LA mixtures is a promising alternative to traditional HMA technologies, reducing environmental impact while ensuring durability and safety. The SMA 8 LA mixture is a better choice than SMA 11 LA due to its performance, providing better overall efficiency and effectiveness.

## Figures and Tables

**Table 1:** The selected properties of tested modified bitumen.

Property	Test Method	PMB 45/80-55	PMB 45/80-55 Clinoptilolite	PMB 45/80-55 Na-P1
Penetration at 25 °C [0.1 mm]	[53]	67.2	61.7	60.7
Softening point R&B [°C]	[54]	65.7	67.9	63.7
Dynamic viscosity at 90 °C [Pa·s]	[55]	113.5	106.7	117.0
Elastic recovery at 25 °C [%]	[56]	86.1	86.3	85.1
Force ductility at 10 °C [J/cm^2^]	[57]	5.73	6.60	7.44
Penetration index [PI]	[58]	2.92	3.07	2.25

**Table 2:** Physical properties of coarse aggregate.

Properties	Test Method	Gabbro
Resistance to polishing *PSV* [-]	[60]	52
Resistance to wear *M_DE_* [%]	[61]	12
Resistance to fragmentation *LA* [%]	[62]	18
Resistance to freezing and thawing *F_NaCl_* [%]	[63]	0.3
Density *ρ_a_* [Mg/m^3^]	[64]	2.966
Water absorption *WA_24_* [%]		0.3

**Table 3:** Aggregate particle size distribution.

Sieves [mm]	Passing Fraction [%]		
	SMA 11	SMA 11 LA	SMA 8 LA
16	100	100	100
11.2	96	95	100
8	56	32	90
5.6	41	19	20
4	-	18	16
2	24	16	15
0.125	10	6	8
0.063	9.5	5.8	8

**Table 4:** Volumetric parameters of SMA and SMA LA reference mixture (HMA).

Parameters	Test Method	SMA 11	SMA 11 LA	SMA 8 LA
Density *ρ_mv_* [Mg/m^3^]	[67]	2.573	2.633	2.633
Bulk density *ρ_bssd_* [Mg/m^3^]	[68]	2.490	2.442	2.354
Air voids *V_m_* [%]	[69]	3.2	10.5	10.6
Voids filled with binder—VFB [%]		82.2	58.7	57.6
Voids in mineral aggregate—VMA [%]		17.9	25.4	25.0
Binder content [%]	-	6.1	6.3	6.3

**Table 5:** Volumetric parameters of SMA LA mixture with the addition of clinoptilolite (WMA).

Parameters	Test Method	SMA 11 LA Clinoptilolite	SMA 8 LA Clinoptilolite
Density *ρ_mv_* [Mg/m^3^]	[67]	2.633	2.633
Bulk density *ρ_bssd_* [Mg/m^3^]	[68]	2.443	2.350
Air voids *V_m_* [%]	[69]	10.4	10.8
Voids filled with binder—VFB [%]		59.0	57.1
Voids in mineral aggregate—VMA [%]		25.3	25.2
Binder content [%]	-	6.3	6.3

**Table 6:** Volumetric parameters of SMA LA mixture with the addition of Na-P1 (WMA).

Parameters	Test Method	SMA 11 LA Na-P1	SMA 8 LA Na-P1
Density *ρ_mv_* [Mg/m^3^]	[67]	2.633	2.633
Bulk density *ρ_bssd_* [Mg/m^3^]	[68]	2.443	2.343
Air voids *V_m_* [%]	[69]	10.4	11.0
Voids filled with binder—VFB [%]		59.0	56.6
Voids in mineral aggregate—VMA [%]		25.3	25.3
Binder content [%]	-	6.3	6.3

**Table 7:** Results of vertical water permeability.

Type of Asphalt Mixture	Q_v_ [m^3^/s]	K*_v_* [m/s]
SMA 8 LA	1.90 × 10^−5^	5.05 × 10^−4^
SMA 8 LA clinoptilolite	1.90 × 10^−5^	4.95 × 10^−4^
SMA 8 LA Na-P1	1.91 × 10^−5^	4.98 × 10^−4^
SMA 11 LA	1.10 × 10^−4^	3.00 × 10^−3^
SMA 11 LA clinoptilolite	1.10 × 10^−4^	3.01 × 10^−3^
SMA 11 LA Na-P1	1.11 × 10^−4^	3.00 × 10^−3^

**Table 8:** Results of horizontal water permeability.

Type of Asphalt Mixture	Q*_h_* [m^3^/s]	K_h_ [m/s]
SMA 8 LA	1.10 × 10^−4^	1.09 × 10^−3^
SMA 8 LA clinoptilolite	1.03 × 10^−4^	1.02 × 10^−3^
SMA 8 LA Na-P1	1.07 × 10^−4^	1.04 × 10^−3^
SMA 11 LA	2.29 × 10^−4^	2.25 × 10^−3^
SMA 11 LA clinoptilolite	2.22 × 10^−4^	2.20 × 10^−3^
SMA 11 LA Na-P1	2.24 × 10^−4^	2.22 × 10^−3^

## Data Availability

The original contributions presented in this study are included in the article, and further inquiries can be directed to the corresponding author.
